# Supplementary material for: APALORD: An R-based tool for differential alternative polyadenylation analysis of long-read RNA-seq data
Source: bioRxiv. 2025 Jun 17:2025.06.11.658931. Preprint. [Version 1] doi: 10.1101/2025.06.11.658931 (PMC12262201; doi:10.1101/2025.06.11.658931)
Supplement: Supplement 1 [file NIHPP2025.06.11.658931v1-supplement-1.pdf]

Figure S1

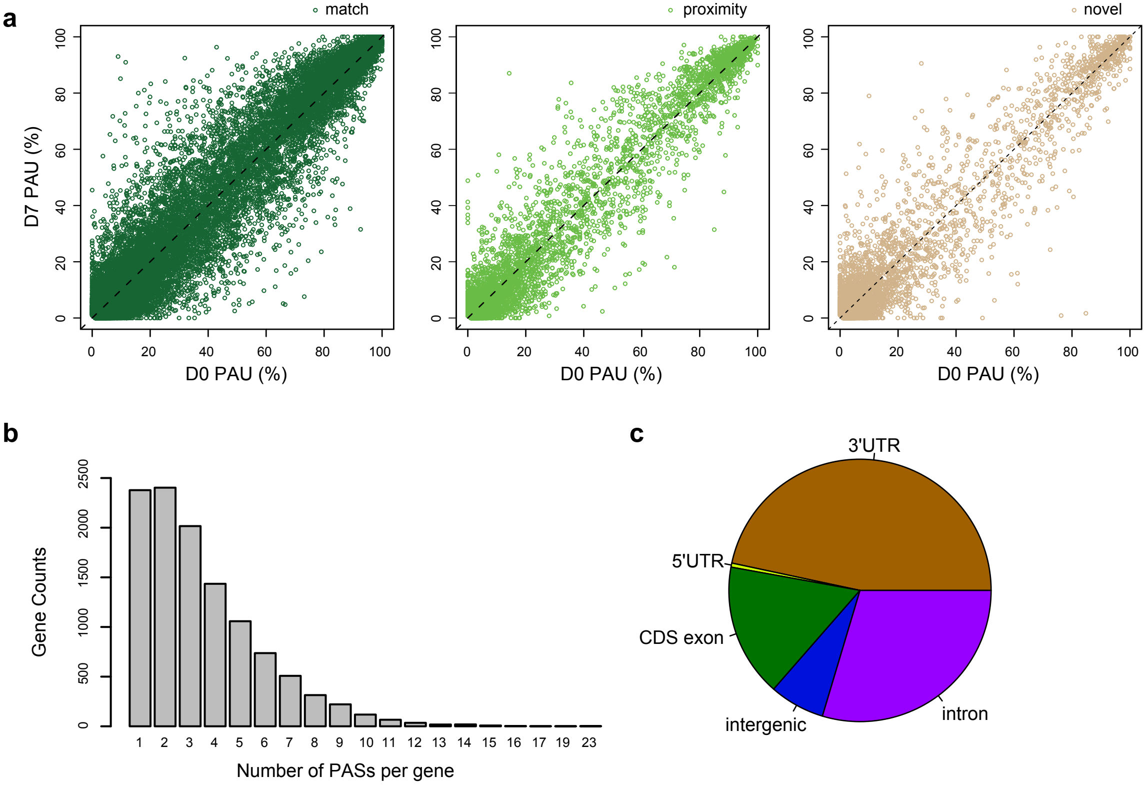
a
match
proximity
novel
D7 PAU (%)
D0 PAU (%)
0
20
40
60
80
100
b
Gene Counts
0
500
1000
1500
2000
2500
1 2 3 4 5 6 7 8 9 10 11 12 13 14 15 16 17 19 23
Number of PASs per gene
c
3'UTR
5'UTR
CDS exon
intergenic
intron

Figure S2

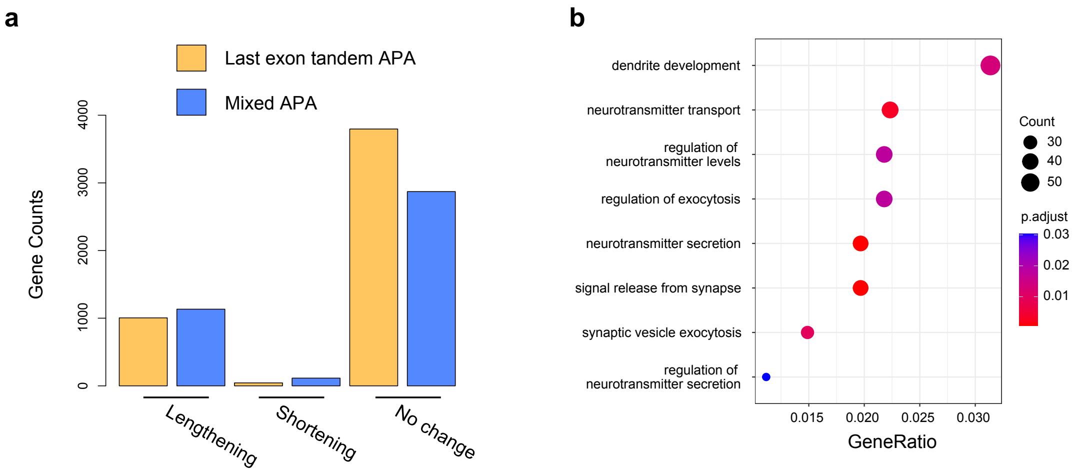
a
Last exon tandem APA
Mixed APA
Gene Counts
0
1000
2000
3000
4000
Lengthening
Shortening
No change
b
dendrite development
neurotransmitter transport
regulation of neurotransmitter levels
regulation of exocytosis
neurotransmitter secretion
signal release from synapse
synaptic vesicle exocytosis
regulation of neurotransmitter secretion
0.015
0.020
0.025
0.030
GeneRatio
Count
30
40
50
p.adjust
0.03
0.02
0.01

Figure S3

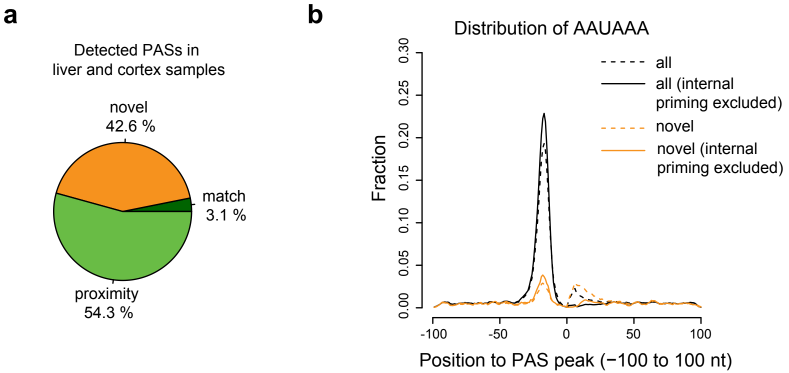
a
Detected PASs in
liver and cortex samples
novel
42.6 %
match
3.1 %
proximity
54.3 %
b
Distribution of AAUAAA
Fraction
0.00
0.05
0.10
0.15
0.20
0.25
0.30
-100
-50
0
50
100
Position to PAS peak (−100 to 100 nt)
all
all (internal
priming excluded)
novel
novel (internal
priming excluded)

Figure S4

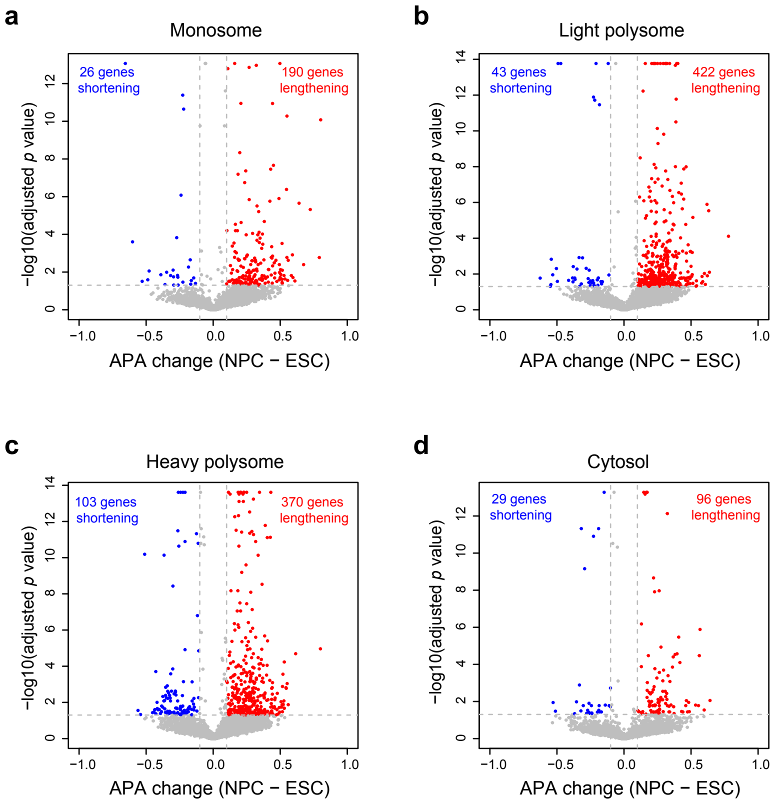
a
Monosome
26 genes shortening
190 genes lengthening
−log10(adjusted p value)
APA change (NPC − ESC)
b
Light polysome
43 genes shortening
422 genes lengthening
−log10(adjusted p value)
APA change (NPC − ESC)
c
Heavy polysome
103 genes shortening
370 genes lengthening
−log10(adjusted p value)
APA change (NPC − ESC)
d
Cytosol
29 genes shortening
96 genes lengthening
−log10(adjusted p value)
APA change (NPC − ESC)

Supplementary Fig.1: APALORD performance in PAS calling from direct RNA-seq of hESCs and hESC-derived neurons.

a-**c** PAU data of match (**a**), proximity (**b**) or novel (**c**) PASs identified by APALORD in hESC (D0) and hESC-derived neuron (D7) samples. match: PASs aligned with PolyA_DB-annotated PASs; proximity: PASs within 20 nt of annotated PASs; novel: PASs outside of match and proximity criteria. **b** Number of genes with varying counts of PASs identified by APALORD in hESC (D0) and hESC-derived neuron (D7) samples. **c** Characterizing the gene body residence of novel PASs identified by APALORD in hESC (D0) and hESC-derived neuron (D7) samples.

Supplementary Fig.2: APA types and GO analysis of neural differentiation-regulated APA genes identified by APALORD.

a Counts of last exon tandem APA and mixed APA genes identified by APALORD, categorized by 3'UTR change during neural differentiation (lengthening, shortening or no change) as defined in Fig.3d. **b** GO analysis of biological processes for genes with significant 3'UTR lengthening in hESC-derived neurons compared to hESCs.

Supplementary Fig.3:

a All the PASs identified by APALORD (without internal priming filtering) from PacBio cDNA data of human cortex and liver, categorized as: match (aligned with annotated PASs in the PolyA_DB reference), proximity (within 20 nt of an annotated PAS), novel (outside match and proximity criteria). **b** Proportion of PASs with the canonical AAUAAA hexamer within the sequence surrounding the peak cleavage site (-100 nt to +100 nt), before or after internal priming filtering. Categories of PASs as defined in **a**.

Supplementary Fig.4:

a-d Transcriptome-wide APA changes quantified by APALORD in NPCs compared ESCs across fractions: monosome polysome (**a**), light polysome (**b**) and heavy polysome (**c**) and cytosol (**d**).
